# Therapeutic Effect of Ultrasound Combined With Porous Lipid Clioquinol/PLGA Microbubbles on Ferroptosis in HL-1 Cardiac Cell Induced by Isoproterenol Attack

**DOI:** 10.3389/fphar.2022.918292

**Published:** 2022-07-22

**Authors:** Nana Li, Lei Dong, Yuanyuan Shen, Yongling Wang, Liansheng Chang, Hongwei Wu, Yuqiao Chang, Menghao Li, Dan Li, Zhaoyi Li, Mei He, Cheng Li, Yao Wei, Haiqin Xie, Feng Wang

**Affiliations:** ^1^ Henan Key Laboratory of Medical Tissue Regeneration, School of Basic Medical Sciences, Xinxiang Medical University, Xinxiang, China; ^2^ National-Regional Key Technology Engineering Laboratory for Medical Ultrasound, School of Biomedical Engineering, Health Science Center, Shenzhen University, Shenzhen, China; ^3^ Department of Physiology and Pathophysiology, School of Basic Medical Sciences, Xinxiang Medical University, Xinxiang, China; ^4^ Department of Human Anatomy, Histology and Embryology, School of Basic Medical Sciences, Xinxiang Medical University, Xinxiang, China; ^5^ Department of Chemistry, Xinxiang Medical University, Xinxiang, China; ^6^ Department of Ultrasound, Peking University Shenzhen Hospital, Shenzhen, China

**Keywords:** ferroptosis, clioquinol, microbubbles, low-frequency ultrasound, cardiovascular diseases

## Abstract

In recent years, studies have shown a close relationship between cardiomyocyte death and ferroptosis. Clioquinol (CQ) can inhibit ferroptosis. Porous lipid-poly (lactic-co-glycolic acid) (PLGA) microbubbles (MBs) were prepared by double emulsification (W_1_/O/W_2_) using 1,2-dioctadecanoyl-sn-glycero-3-phophocholine and PLGA as raw materials. Porous lipid-PLGA MBs were used as carriers to prepare CQ/PLGA MBs containing CQ. CQ/PLGA had the advantages of high drug loading, good biocompatibility, and sustained release. Our results showed that CQ/PLGA improved the effect of CQ and reduced its cytotoxicity. Under low-frequency ultrasound with certain parameters, CQ/PLGA showed steady-state cavitation, which increased the membrane permeability of mouse cardiomyocyte HL-1 to a certain extent and further prevented the process of ferroptosis in mouse cardiomyocyte HL-1.

## Introduction

According to public data from the World Health Organization, noncommunicable diseases (NCDs) account for 71% of all deaths (Lowe, 2020). Cardiovascular and cerebrovascular diseases are the leading causes of death, accounting for 44% of all deaths due to NCDs, twice as many as cancer deaths (Lowe, 2020). The total number of deaths from cardiovascular diseases is expected to exceed 23 million by 2030(Birn and Nervi, 2020). On the whole, the prevalence and mortality of cardiovascular diseases are still on the rise. Especially in recent years, the mortality rate of cardiovascular diseases in rural areas has become higher than that in urban areas (Zhou and Bei, 2020). A rapid increase in the total cost of hospitalization due to cardiovascular and cerebrovascular diseases has increased the burden of cardiovascular diseases, making them a major public health problem. Cardiovascular diseases should urgently be prevented and cured. Therefore, the pathogenesis of cardiovascular diseases, the mechanism of cardiomyocyte death, and intervention methods should immediately be studied (Zhou and Bei, 2020; Piccoli, 2021).

Recent studies have shown that ferroptosis is closely related to cardiomyocyte death (Li et al., 2020; Ma et al., 2020; Wu et al., 2021). Ferroptosis is a mode of regulatory cell death. The hypothesis of “siderogenic heart disease” was formally put forward by Sullivan in 1981 and was named by Dixon et al. for the first time in 2012 (Li et al., 2020). At present, the mechanism of ferroptosis is thought to be related to amino acid metabolism, iron metabolism, and lipid metabolism (Wu et al., 2021). The initiation and execution of ferroptosis are based on the intersection of amino acid metabolism, fat metabolism, and iron metabolism, which results from the disturbance of phospholipid oxidation metabolism in the cell membrane. When lipid peroxides accumulate to the limit of glutathione peroxidase reduction, iron ion-mediated Fenton reaction catalyzes the production of lipid-free radicals. A large number of lipid-free radicals accumulate in cells and lead to cell death (Chen et al., 2021).

Clioquinol (CQ), with the chemical formula C_9_H_5_C_l_IN_O_, was used to treat diseases, such as dysentery, in the 20th century and has been found to regulate the dynamic balance of Fe^2+^ ions in tissue through their effective chelation (Lv et al., 2021). The clinical application of CQ is seriously limited because of its poor water solubility, low bioavailability, and acute cytotoxicity (Khan et al., 2020).

Microbubbles (MBs) have a variety of therapeutic use. For example, they are used as contrast agents in ultrasound (US) to improve their diagnostic efficacy (Luo et al., 2017). However, conventional lipid-based MBs have some disadvantages such as poor drug encapsulation, weak drug loading efficiency, and weak drug release ability (Luo et al., 2017; Rix et al., 2020; Klibanov, 2021). Chen et al. developed a new type of porous lipid-poly (lactic-co-glycolic acid) (PLGA) MBs (lipid/PLGA MBs), which solved the dilemma of MBs as imaging agents and drug carriers (Chen et al., 2019; Wang et al., 2021). When irradiated with US with appropriate energy, lipid/PLGA MBs oscillated rapidly and changed the size of MBs periodically (Chen et al., 2019). The oscillation of lipid/PLGA MBs might produce stable cavitation and inertial cavitation to increase the permeability of the cell membrane, thus promoting the transmembrane drug delivery into the cell.

**FIGURE 1:**
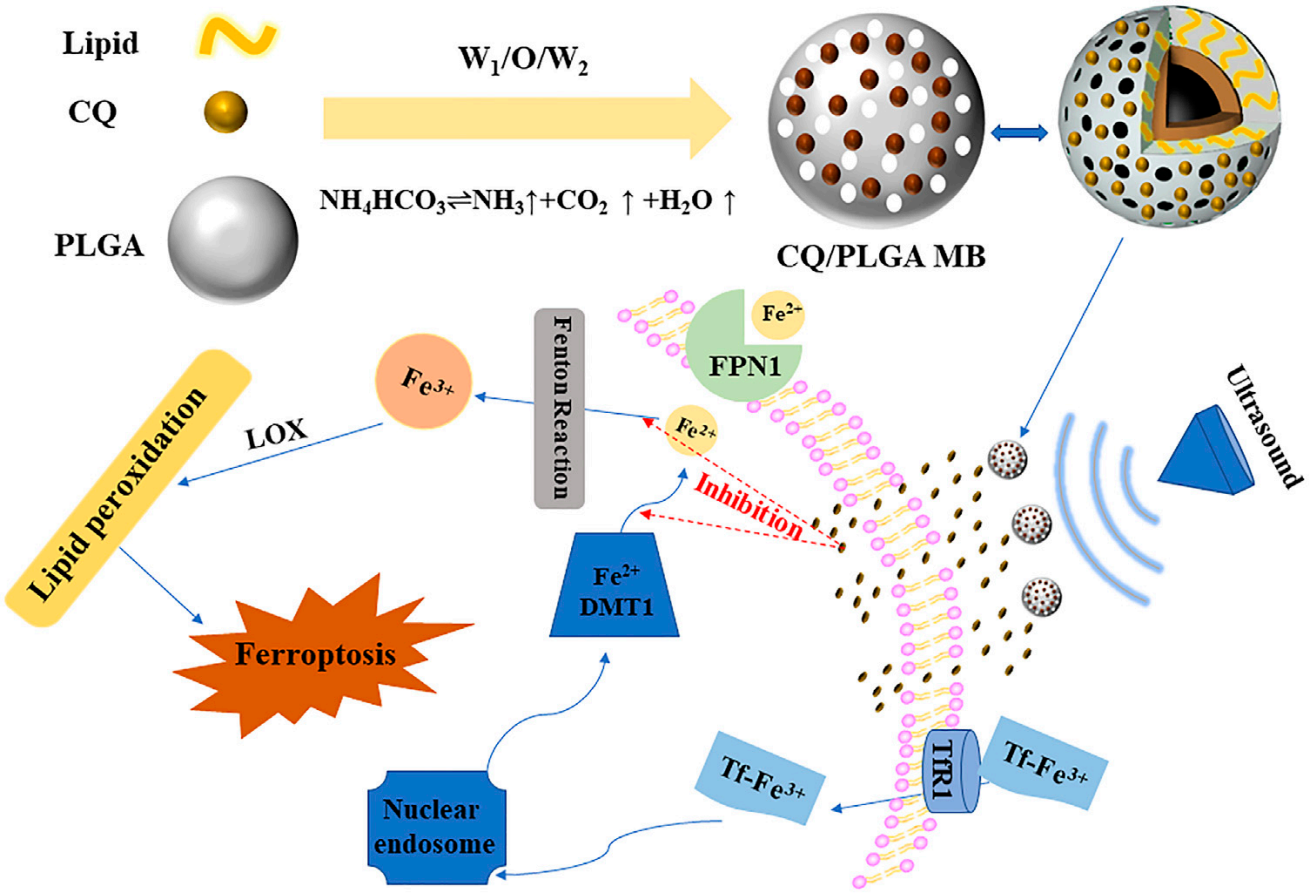
Ultrasound combined with CQ/PLGA MBs inhibited the ferroptosis pattern of mouse cardiomyocytes. Under the action of ultrasound, the “steady-state cavitation effect” of CQ/PLGA MBs caused the increase in cell membrane permeability of HL-1 cells in a short period of time. At the same time, CQ/PLGA releases CQ, and CQ enters the cell through the open space of the cell membrane and binds to Fe^2+^ ions, blocking the occurrence of ferroptosis in HL-1 cells.

Based on these facts, we synthesized porous lipid/PLGA hybrid MBs (CQ/PLGA MBs) containing CQ while testing the characterization of CQ/PLGA, including drug loading, entrapment efficiency, and particle size potential distribution. Ferroptosis in HL-1 of mouse cardiomyocytes was induced using erastin, which verified the effect of CQ in blocking ferroptosis in HL-1. CQ had certain cytotoxicity. In addition, isoproterenol (ISO) could induce ferroptosis in HL-1 cells, which could be blocked by CQ. We further explored the inhibitory effect of CQ/PLGA on ferroptosis in HL-1 and the ultrasonic sensitivity of CQ/PLGA. Finally, our results showed that CQ could block the occurrence of ferroptosis in HL-1. Under the action of US, the “steady-state cavitation effect” of CQ/PLGA MBs caused an increase in the cell membrane permeability of HL-1 cells in a short period of time. At the same time, CQ/PLGA released CQ, and CQ entered the cell through the open space of the cell membrane and bound to Fe^2+^ ions, blocking the occurrence of ferroptosis in HL-1 cells (Figure 1). The better inhibition of the process of ferroptosis in HL-1 is expected to provide a new scheme for the treatment of cardiovascular diseases.

## Materials and Methods

### Synthesis of Clioquinol/PLGA MBs

1,2-Dioctadecanoyl-sn-glycero-3-phophocholine (DSPC, 0.0025 g, Avanti polar lipids Inc, United States) and 0.05 g of PLGA (Guangzhou Cellcook Biotech, Guangzhou, China) were dissolved in 1 ml of dichloromethane to form a colorless transparent solution. NH_4_HCO_3_ (0.06 g) was dissolved in 1 ml of double-distilled water, and 200 μl of the mixture was extracted into the aforementioned solution. The obtained mixed solution was emulsified using an ultrasonic cell pulverizer (SCIENTZ-1200E, Ningbo Scientz Biotechnology, Ningbo, China) with a power of 650 W (the trigger interval was 3 s, the power ratio was 45%, and the horn was Φ 6). A 4% PVA solution (5 ml) was added to the mixed solution, and the solution was homogenized. Double-distilled water (10 ml) was added to the solution obtained previously, mixed well, stirred for 2.5–3 h, and centrifuged three times (4500 rpm, 5 min). The supernatant was poured into 1 ml of double-distilled water and frozen for 3–4 h in a refrigerator at −80°C. The freeze-dried powder of porous lipid-PLGA MBs was obtained after freeze-drying in a vacuum freeze-dryer for about 24 h. The obtained powder was stored in a refrigerator at 4°C and set aside. When preparing CQ/PLGA lipid MBs, a certain quality of CQ (Shanghai Aladdin Biochemical Technology, Shanghai, China) could be dissolved in a dichloromethane solution.

### Characterization of Clioquinol/PLGA MBs

CQ/PLGA was dissolved in dimethyl sulfoxide. The CQ absorption at 322 nm wavelength was measured using an ultraviolet spectrophotometer (GENESYS 10S UV–vis spectrophotometer, Thermo Scientific, MA, United States). Under the above wavelength, the drug efficiency and drug entrapment efficiency of CQ/PLGA were detected using an enzyme labeling instrument (BioTek, Winooski, VT, United States). Then, the drug loading and entrapment efficiencies of CQ/PLGA dissolved in dimethyl sulfoxide were calculated. Drug loading was calculated as LE (%) = We/Wm × 100%, where LE is the drug loading efficiency, W_e_ is the amount of drug encapsulated in the lipid, and W_m_ is the total weight of CQ/PLGA MBs. Entrapment efficiency was calculated as EN (%) = (1 − C_f_/C_t_) × 100, where EN is the drug entrapment efficiency as a percentage, C_f_ is the amount of free drug, and C_t_ is the total amount of drug (free and entrapped). The particle size of CQ/PLGA was measured using a particle size analyzer (Zetasizer Nano, Malvern Instruments, Malvern, United Kingdom). The shape of CQ/PLGA was observed using a scanning electron microscope (SEM, Akishima, Tokyo, Japan). We tested the stability of CQ/PLGA MBs. The particle size and concentration of CQ/PLGA MBs were measured at the initial, 1st, 2nd, 4th, 6th, and 8th days. The concentration of porous lipid CQ/PLGA MBs is expressed by OD_500_.

### Cell Cultures

Cardiomyocyte HL-1 cells were purchased from Shanghai Cell Bank (Shanghai, China). Fetal bovine serum (FBS), penicillin–streptomycin (PS), and trypsin were purchased from Gibco (CA, United States). HL-1 cells were cultured in a conventional humidified CO_2_ incubator at 37°C. These cells were routinely cultured in a high glucose medium (Dulbecco’s Modified Eagle Medium) supplemented with 10% FBS and 1% PS. The culture medium was changed every 2 days, and the cell fusion degree reached 80%.

### Cell Viability Assay (CCK-8)

Cell viability was determined using the CCK-8 assay (Dojindo Laboratories, Shanghai, China). The cell suspension was inoculated in a 96-well plate (100 μl per well), and the same sample could be repeated three times. After different treatments, the culture plate was put into an incubator for 24 h (37°C, 5% CO_2_). CCK-8 solution (10 μl) was added to each hole, and the culture plate was put into an incubator for 1–4 h. The absorbance value (OD) at 450 nm was measured using an enzyme labeling instrument. Cell viability (%) = [A (1) − A (blank)]/[A (0) − A (blank)] × 100. A (1): OD values of pores with cells, CCK-8 solutions, and drug solutions. A (0): OD value of pores with cell and CCK-8 solution but no drug solution. A (blank): OD value of hole without cells.

### Cytotoxicity Evaluation of Clioquinol

HL-1 Cells were seeded at a density of 5 × 104 cells/ml in a 96-well plate (100 μl per well). When the degree of cell fusion was 80%, the medium containing 0, 10, 20, 40, 60, 80, and 100 μg/ml CQ was replaced. After 24 h, the cell state was observed under a microscope, and the cell viability of HL-1 cells corresponding to each concentration of CQ was detected using CCK-8.

### US-Guided Drug Release

The maximum ultraviolet absorption peak of CQ detected using an ultraviolet spectrophotometer was 322 nm. Under 322 nm, the OD value of CQ/PLGA suspension before and after ultrasonication was detected using an enzyme labeling instrument (96-hole plate, 100 μl per hole, and five compound holes). The release amount of CQ in the solution was calculated. The center frequency of US was 949 kHz; the pulse interval was 1 s; the pulse cycle was 10,000; the intensity of US was 60, 80, 100, 110, 120, and 130 mvpp; and the duration was 60 s. When the US intensity was 100 mvpp, the shape changes in CQ/PLGA before and after US were observed using an SEM after continuous action for 60 s.

### Occurrence and Intervention of Ferroptosis in HL-1

As mentioned previously, HL-1 cells were inoculated in 96-well plates. When the degree of cell fusion was 80%, HL-1 cells were incubated with erastin, a ferroptosis inducer at the concentrations of 0, 2, and 4 μM, for 24 h. ISO was obtained from Cayman Chemical (Cayman Chemical, Ann Arbor, MI, United States). ISO, an inducer of cardiomyocyte apoptosis, was incubated with HL-1 cells for 24 h. The HL-1 cells were incubated with 4-μM erastin medium or 2-mM ISO medium and CQ at 0, 10, 20, 40, 60, 80, and 100 μg/ml (control group without erastin and CQ) for 24 h. The viability of cells was detected using the CCK-8 reagent, and the survival status of cells was observed under a microscope.

### Propidium Iodide Fluorescence Apoptosis Detection

An Annexin V-FITC/PI double-stained apoptosis detection kit was purchased from Keygen Biotech Co. (Nanjing, China). Similar to that mentioned above, HL-1 cells were inoculated in 96-well plates. When the cell fusion was 80%, the cells underwent five different treatments: the control group did not undergo any treatment, the ISO group was treated with ISO, the CQ group was incubated with ISO and CQ, the CQ/PLGA group was incubated with ISO and CQ/PLGA, and the US + CQ/PLGA group was incubated with ISO and CQ/PLGA. The ultrasonic treatment was carried out at the same time. The concentration of ISO was 2 mM, and the final concentration of CQ was 80 μg/ml. The ultrasonic intensity was 100 mvpp, and other ultrasonic parameters were the same as above. 4′,6-Diamidino-2-phenylindole labeled nucleus. PI was used to label apoptotic cells. Phosphate-buffered saline was used to wash the dye. Fluorescence brightness was observed using a fluorescence microscope (Leica; Berlin, Germany). ImageJ (ImageJ, Marlyand, United States).

### Data Analysis

All data were statistically analyzed using GraphPad Prism 6.0 (GraphPad Prism 6.0 GraphPad www.graphpad.com) and SPSS 25.0 (IBM SPSS Statistics for Windows, Version 25.0; Armonk, NY, United States) software. The measurement data were expressed as mean value ± standard deviation (x ± SD). Comparisons between multiple groups were performed using one-way analysis of variance. *p* < 0.05 was considered statistically significant (*p < 0.05, **p < 0.01, ***p < 0.001, ^#^ *p* < 0.05, ^##^ *p* < 0.01, ^###^ *p* < 0.001).

## Results

### Characterization of Lipid Clioquinol/PLGA MBs

**FIGURE 2:**
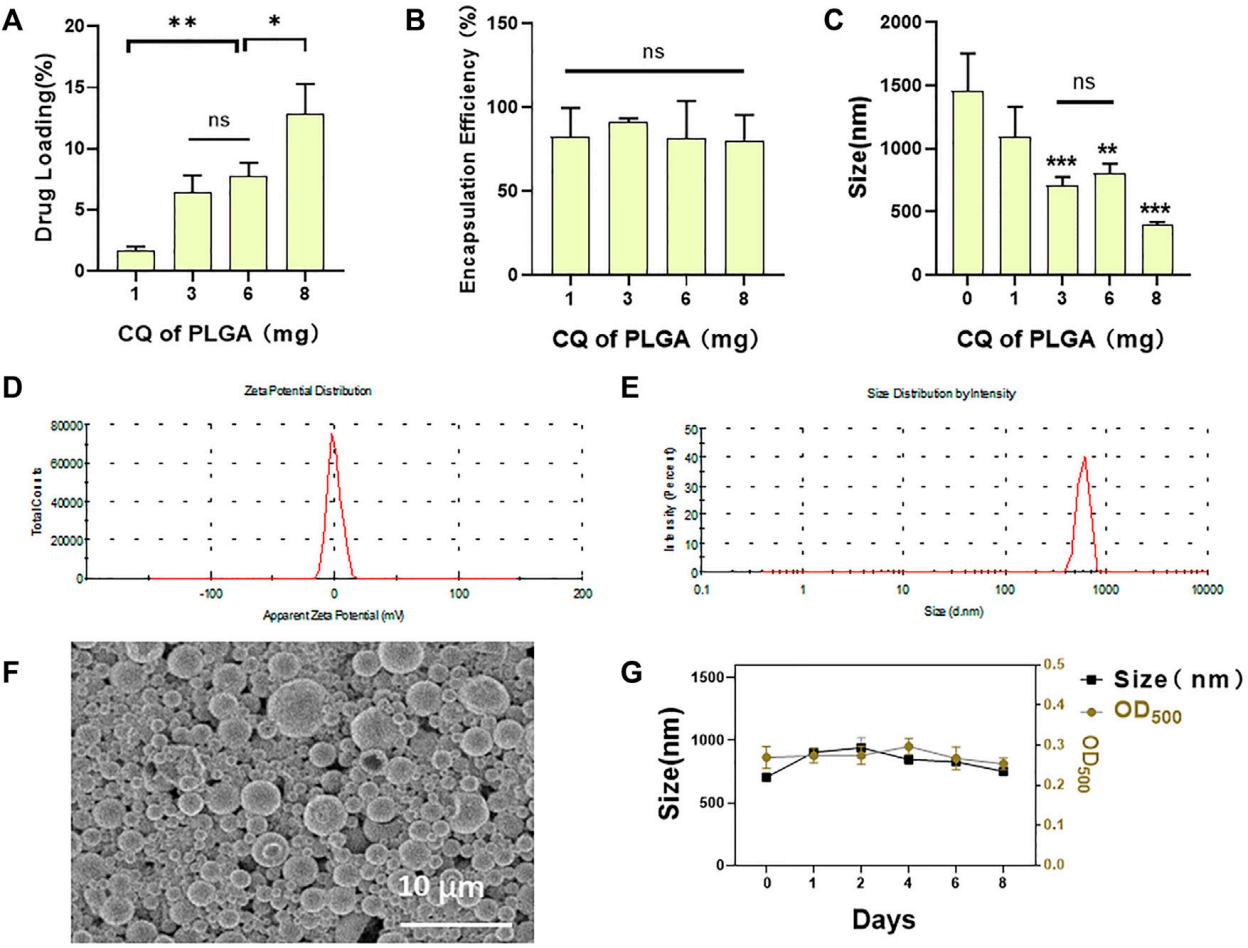
Characterization of CQ/PLGA. **(A)** The drug loading of CQ/PLGA prepared by adding 1-, 3-, 6-, and 8-mg CQ per 50-mg PLGA (comparison between groups, *p < 0.05, **p < 0.01). **(B)** The entrapment efficiency of CQ/PLGA prepared by adding 1-, 3-, 6-, and 8-mg CQ per 50-mg PLGA. **(C)** Particle size distribution of CQ/PLGA prepared by adding 1-, 3-, 6-, and 8-mg CQ per 50-mg PLGA (vs. “0-mg group,” **p < 0.01, ***p < 0.001, *n* = 5). **(D)** The average particle size of CQ/PLGA MBs prepared by adding 6-mg CQ to 50-mg PLGA is 801.78 ± 69.10 nm. **(E)** The average potential of the particle size distribution of CQ/PLGA MBs prepared by adding 6-mg CQ to 50-mg PLGA is −6.57 ± 0.15 mV. **(F)** CQ/PLGA nanoparticles were prepared by adding 6 mg CQ to every 50 mg PLGA. SEM micrographs of nanoparticles showing the shape and the surface characteristics of CQ/PLGA. **(G)** The particle size and concentration of porous lipid CQ/PLGA MBs were measured at the initial, 1st, 2nd, 4th, 6th, and 8th days. The concentration of porous lipid CQ/PLGA MBs is expressed by OD_500_.

CQ/PLGA MBs were prepared by adding 6 mg of CQ to every 50 mg of PLGA. The characterization of CQ/PLGA has been tested (Figure 2A). The drug loading of CQ/PLGA was 9.01% ± 2.34% (Figure 2A) (vs. “8-mg group,” *p < 0.05, vs. “1-mg group,” **p < 0.01). The entrapment efficiency of CQ/PLGA was 75.03% ± 19.46% (Figure 2B). The results of the Malvern particle size analyzer showed that the particle size of CQ/PLGA was 801.78 ± 69.10 nm (vs. “0-mg group,” *p < 0.05), and the potential distribution was 6.57 ± 0.15 mV (Figures 2D,E). Scanning electron microscopy showed that CQ/PLGA was spherical and uniformly dispersed (Figure 2F). CQ/PLGA was found to have good stability by continuously monitoring the particle size and concentration of CQ/PLGA microbubbles for 8 days (Figure 2G). Therefore, we prepared CQ/PLGA MBs, explored the optimum ratio of CQ to PLGA, and tested their characterization.

### Clioquinol Cytotoxicity Assay and Ultrasound-Assisted Drug Release

**FIGURE 3:**
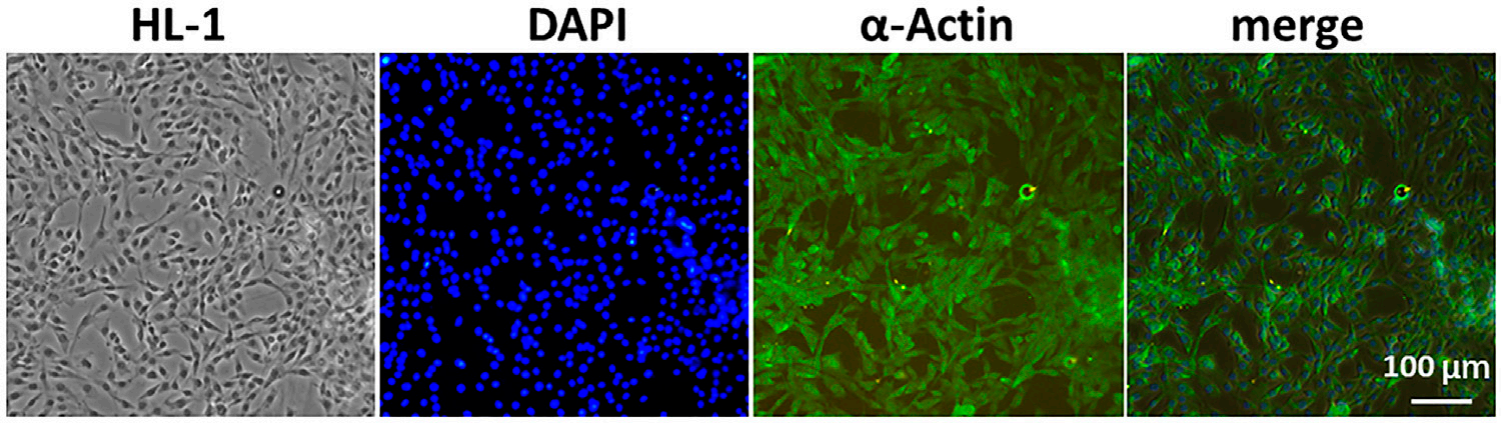
Mouse cardiomyocytes (HL-1 cells) were identified using immunofluorescence. We selected *α*-actin protein highly expressed in HL-1 cells for green fluorescence labeling and then verified the accuracy of mouse cardiomyocytes. DAPI labeled the nucleus. Bar = 100 µm.

**FIGURE 4:**
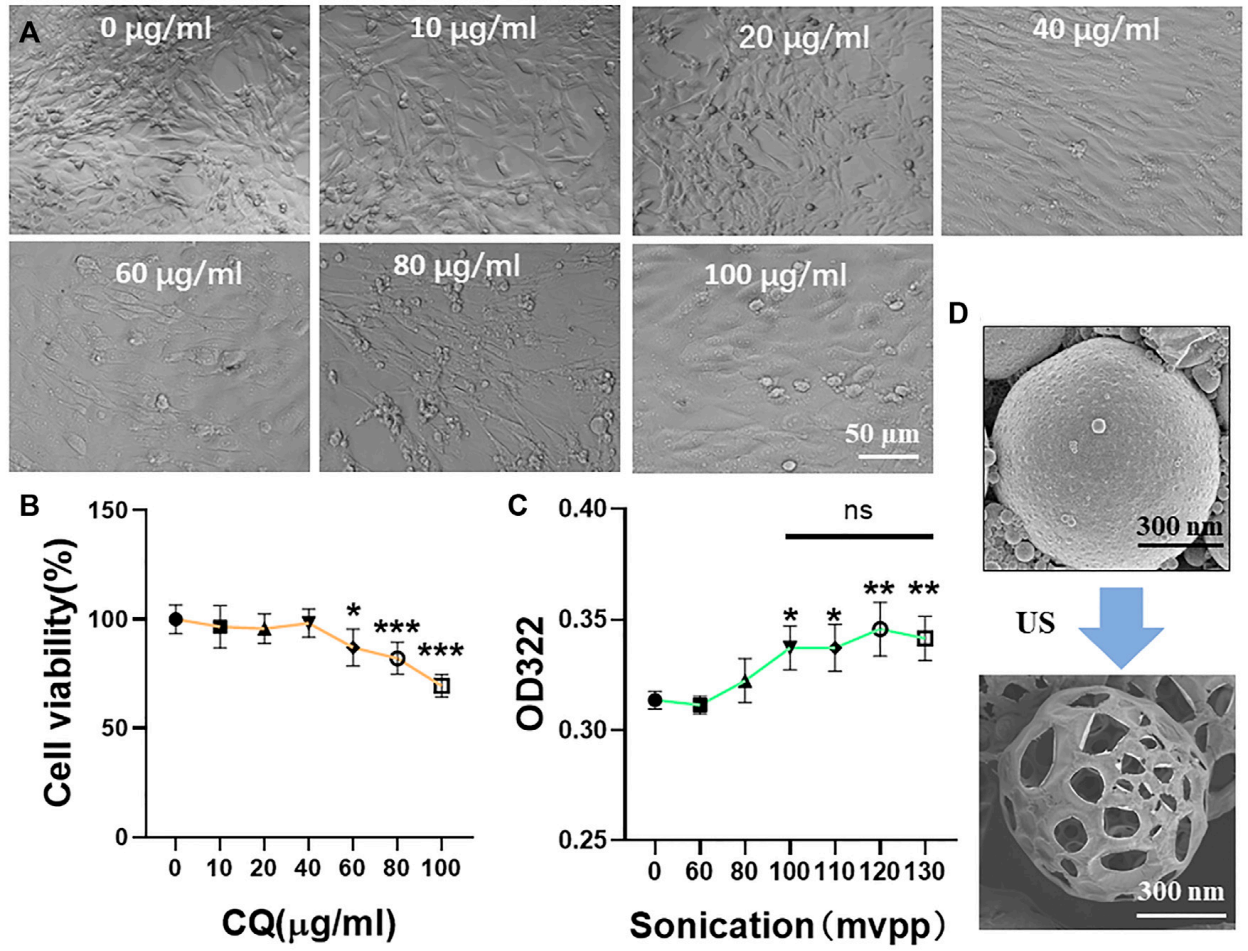
Cytotoxicity test of CQ and ultrasound-assisted drug release. **(A,B)** HL-1 cells were incubated with a medium containing 0, 10, 20, 40, 60, 80, and 100 μg/ml CQ for 24 h to detect the cell viability and observe the cell survival state under a microscope. When the concentration of CQ was 60–100 μg/ml, HL-1 had obvious cytotoxicity (VS. “0-µg/ml group,” *p < 0.05, ***p < 0.001, *n* = 5, Bar = 50 µm). When the concentration of CQ was 60 μg/ml, the viability of HL-1 cells was 87.9% ± 7.73%. **(C)** The maximum UV absorption peak of CQ detected using a UV spectrophotometer was 322 nm. The release of CQ in 100 μl CQ/PLGA suspension was detected after continuous action of different ultrasonic intensities for 60 s. When the ultrasound intensity was 100–130 mvpp, CQ/PLGA was triggered to release CQ (vs. “0-mvpp group,” *p < 0.05, **p < 0.01, *n* = 5). **(D)** When the ultrasound intensity was 100 mvpp, the shape changes in CQ/PLGA before and after ultrasound were observed using a SEM after continuous action for 60 s.

We selected a highly expressed *α*-actin protein in HL-1 cells for green fluorescence labeling. The results showed that the green fluorescence signal was strong, which verified the accuracy of mouse cardiomyocytes. Mouse cardiomyocytes (HL-1 cells) were identified using immunofluorescence (Figure 3). The cytotoxicity of CQ and US-assisted drug release was tested (Figure 4B). HL-1 cells were coincubated with 60 μg/ml CQ for 24 h. The cell viability detected using the CCK-8 reagent was 87.9% ± 7.73% (vs. “0-μg/ml group,” *p < 0.05). The survival state of HL-1 cells was observed under a microscope. The results showed that when the concentration of CQ was 60–100 μg/ml, the volume of HL-1 cells was increased, the density was reduced, and the survival condition was gradually deteriorated (Figure 4A). At 322 nm, the drug release from CQ/PLGA was obvious after 60 s of treatment with a US of 100 mvpp intensity, and the OD value was 0.337 ± 0.01 (vs. “0-mvpp group,” *p < 0.05; Figure 4C). When the US intensity was 100 mvpp, the shape changes in CQ/PLGA before and after US were observed using an SEM after continuous action for 60 s. The results show that CQ/PLGA evolves from spherical to a large number of voids on the surface, that is, the “cavitation effect” occurs and CQ is released (Figure 4D). Therefore, CQ had certain cytotoxicity and could trigger CQ/PLGA to release CQ under the action of US with certain parameters.

### Occurrence of Ferroptosis in HL-1 Cells

**FIGURE 5:**
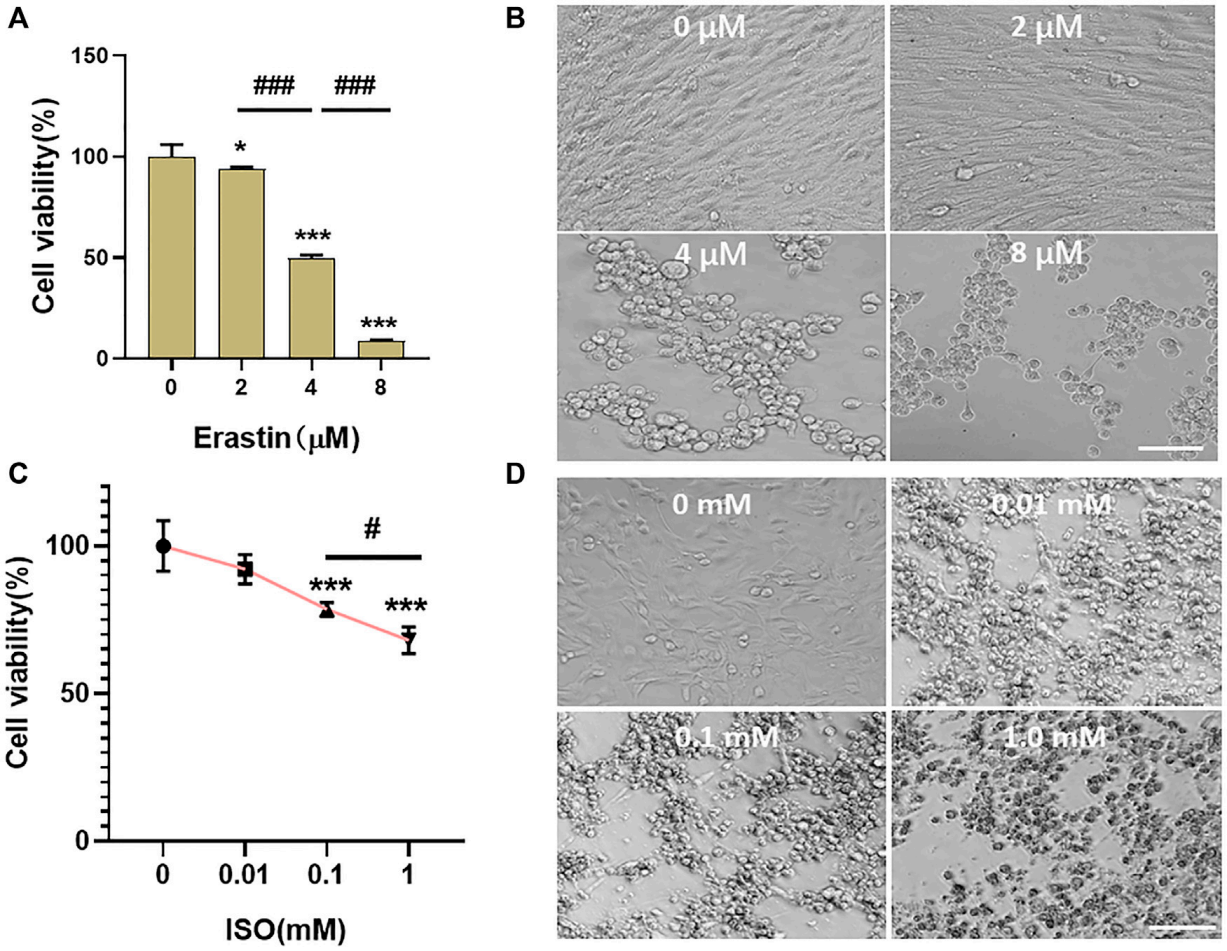
Ferroptosis occurred in HL-1 of mouse cardiomyocytes. **(A,B)** Erastin was incubated with HL-1 cells at concentrations of 0, 2, 4, and 8 μM for 24 h. The viability of cells was detected using the CCK-8 reagent, and the survival status of cells was observed under a microscope. When the concentration of erastin was 4 μM, the cell viability of HL-1 cells was 49.53% ± 1.63% (vs. “0-µM group,” ***p < 0.001, *n* = 5, Bar = 50 µm). **(C,D)** ISO was incubated with HL-1 cells at concentrations of 0, 0.01, 0.1, and 1.0 mM for 24 h. The viability of cells was detected using the CCK-8 reagent, and the survival status of cells was observed under a microscope. When the concentration of ISO was 0.1 mM, the cell viability of HL-1 cells was 78.52% ± 2.13% (vs. “0-µM group,” ***p < 0.001, *n* = 5, Bar = 50 µm).

A classic ferroptosis inducer, erastin, was coincubated with HL-1 cells for 24 h at the concentration of 4 μM (Figure 5). The results showed that the cell viability of HL-1 cells was 49.53% ± 1.63% (Figure 5A) (vs. “0-µM group,” *p < 0.001). Microscopic investigation showed that the cells condensed together and lost their original spindle shape (Figure 5B). ISO was coincubated with HL-1 cells for 24 h at the concentration of 0.1 mM. The viability of HL-1 cells was 78.52% ± 2.13% (vs. “0-µM group,” ***p < 0.001, Figure 5C). Microscopic investigation showed that the state of HL-1 cells induced by erastin was consistent with that of ferroptosis (Figure 5D). Therefore, the mode of cardiomyocyte death was considered to be ferroptosis, and ISO could induce ferroptosis in HL-1 cells.

### Clioquinol Inhibits Ferroptosis in HL-1 Cells

**FIGURE 6:**
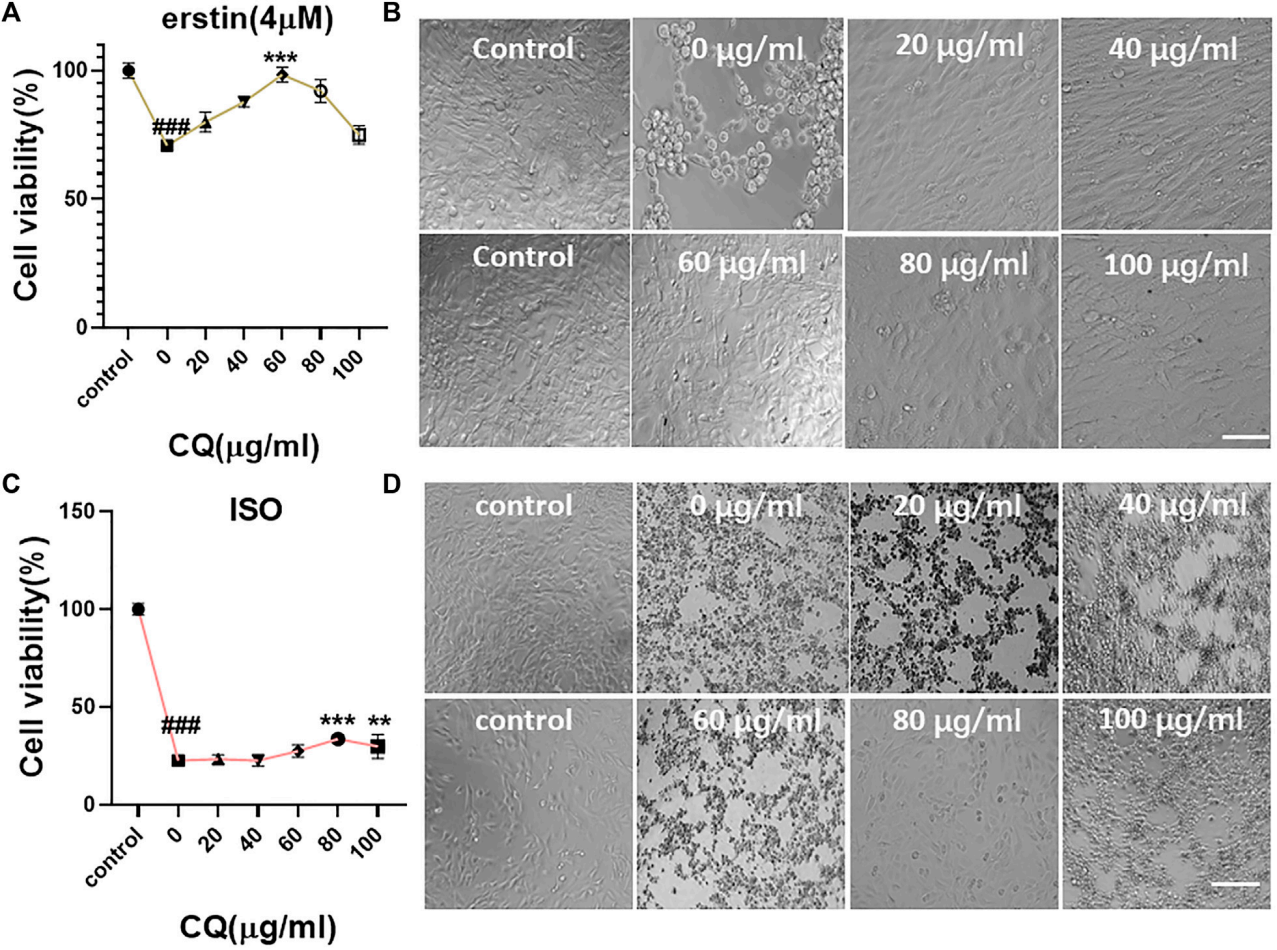
Blocking effect of CQ on ferroptosis in mouse cardiomyocytes. **(A,B)** The medium containing 4 μM erastin and different concentrations of CQ (control group without erastin and CQ) were coincubated with HL-1 cells for 24 h. The viability of cells was detected using the CCK-8 reagent, and the survival status of cells was observed under a microscope. When the concentration of CQ was 0 μg/ml, the cell viability was 70.79% ± 1.91%. When the concentration of CQ was 60 μg/ml, the cell viability was 98.42% ± 2.65% (vs. control group, ###*p* < 0.001, vs. “0-µM group,” ***p < 0.001, *n* = 5, Bar = 50 µm). **(C,D)** The medium containing 2-mM ISO and different concentrations of CQ (control group without ISO and CQ) were coincubated with HL-1 cells for 24 h. The viability of cells was detected using the CCK-8 reagent, and the survival status of cells was observed under a microscope. When the concentration of CQ was 0 μg/ml, the cell viability was 22.52% ± 1.18%. When the concentration of CQ was 80 μg/ml, the cell viability was 33.56% ± 1.84% (vs. control group, ###*p* < 0.001, vs. “0-µM group,” ***p < 0.001, *n* = 5, Bar = 50 µm).

Ferroptosis in HL-1 cells was induced using a 4-μM erastin medium (Figure 6). The results showed that the occurrence of ferroptosis in HL-1 cells could be blocked to some extent when the concentration of CQ was 60 μg/ml. The cell viability was 98.42% ± 2.65% (Figure 6A ) (vs. “0-μM group,” ***p < 0.001; control group without erastin and CQ). Microscopic investigation showed that the cells were evenly distributed and spindle-shaped (Figure 6B). Ferroptosis in HL-1 cells was induced using the ISO medium at the concentration of 2 mM. When the concentration of CQ was 80 μg/ml, the cell viability was 33.56% ± 1.84% (vs. “0-μM group,” ***p < 0.001, Figure 6C). Microscopic investigation showed that the cells were evenly distributed and close to the normal cell state (Figure 6D). Therefore, we verified that CQ could block the occurrence of ferroptosis in HL-1 cells and explored the optimal concentration.

### Evaluation of the Effect of US Combined With Clioquinol/PLGA in Inhibiting Ferroptosis in HL-1

**FIGURE 7:**
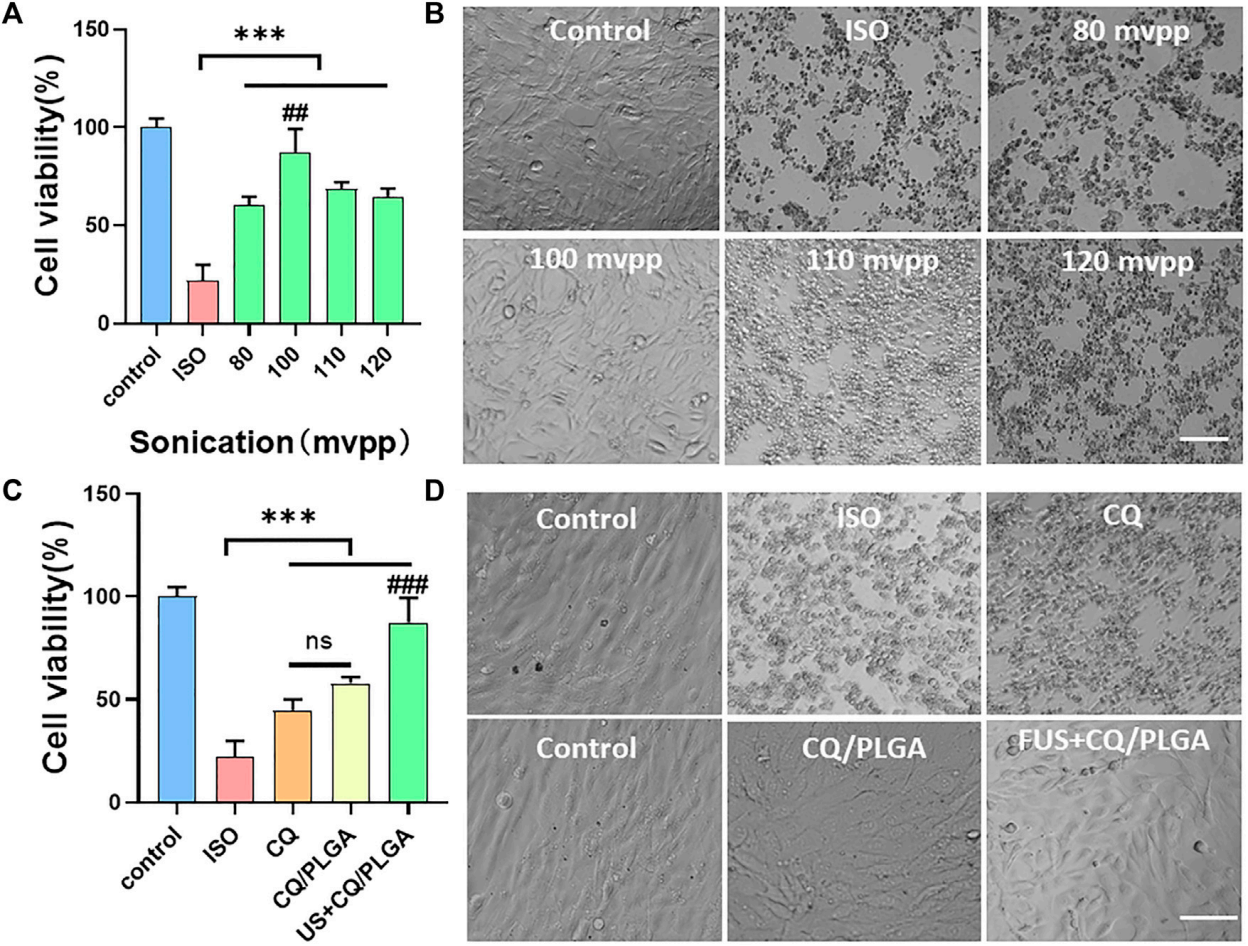
Evaluation of the inhibitory effect of ultrasound combined with CQ/PLGA on ferroptosis in HL-1 via CCK-8 detection. **(A,B)** When the fusion degree of HL-1 cells reaches 80%, the culture medium containing ISO (concentration of 2 mM) and CQ/PLGA (concentration of final drug of CQ is 80 μg/ml) is added and treated with ultrasound for 30 s (ultrasound intensity is 0,80,100,110, and 120 mvpp), in which the control group does not do any treatment. After 24 h of incubation, the viability of cells was detected using the CCK-8 reagent, and the survival status of cells was observed under a microscope. When the intensity of ultrasound is 100 mvpp, ultrasound combined with CQ/PLGA has the best effect on inhibiting the occurrence of ferroptosis in HL-1. The cell viability was 87.39% ± 10.64% (vs. “ISO group,” ***p < 0.001, vs. other groups, ##*p* < 0.01, *n* = 5, Bar = 50 µm). **(C,D)** When the fusion degree of HL-1 cells reached 80%, the HL-1 cells were treated with different treatments and incubated for 24 h. The results showed that ultrasound combined with CQ/PLGA could better inhibit the ferroptosis process of HL-1 caused by ISO. The cell viability of the ISO group was 22.11% ± 7.02%, and the cell viability of ultrasound combined with the CQ/PLGA group was 83.16% ± 7.21% (vs. “ISO group,” vs. other groups, ###*p* < 0.001, *n* = 5, Bar = 50 µm).

Ferroptosis in HL-1 cells was induced by the ISO medium at the concentration of 2 mM. The final concentration of CQ was 80 μg/ml (Figure 7). The viability of HL-1 cells in the CQ/PLGA group was 57.92% ± 2.57% (vs. the ISO group, ***p < 0.001). When the ultrasonic intensity was 100 mvpp, the US combined with CQ/PLGA had the best inhibitory effect on ferroptosis in HL-1. The cell viability was 87.39% ± 10.64% (Figure 7A) (vs. the ISO group, ***p < 0.001, vs. other groups, ^##^ *p* < 0.01). Microscopic investigation showed that the cells were evenly distributed and close to the normal cell state (Figure 7B). Compared with CQ, CQ/PLGA group, US combined with CQ/PLGA could better inhibit the ferroptosis process of HL-1 caused by ISO. The corresponding cell viability of the US + CQ/PLGA group was 83.16% ± 7.21% (vs. the ISO group, ***p < 0.001, vs. other groups, ^###^ *p* < 0.001, Figure 7C). Microscopic investigation showed that the cells were evenly distributed in the US combined with CQ/PLGA treatment group, which was close to the normal cell state (Figure 7D).

**FIGURE 8:**
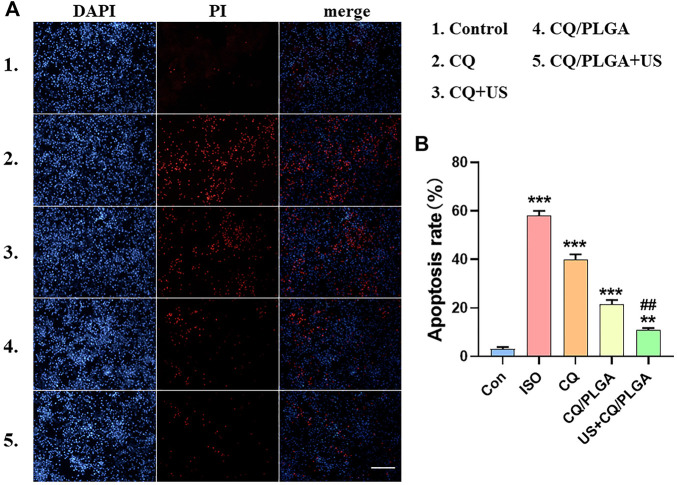
Evaluation of the inhibitory effect of ultrasound combined with CQ/PLGA on ferroptosis in HL-1 using fluorescent apoptosis detection. **(A,B)** When the fusion degree of HL-1 cells reached 80%, HL-1 cells were treated with different treatments (the control group did not do any treatment). After incubation for 24 h, the nuclei were counterstained with PI. The results of ImageJ analysis showed that ultrasound combined with CQ/PLGA could inhibit the apoptosis of HL-1 induced by ISO. The apoptosis rate of the ISO group was 58.08% ± 1.58%, and the apoptosis rate of the US + CQ/PLGA group was 10.90% ± 0.68% (vs. control group, **p < 0.01, ***p < 0.001, vs. other groups, ##*p* < 0.01, *n* = 5, Bar = 50 µm).

The inhibitory effect of US combined with CQ/PLGA on ferroptosis in HL-1 using fluorescent apoptosis detection was evaluated (Figure 8). The results of the PI apoptosis fluorescence detection showed that the apoptosis rate of HL-1 cells in the ISO group was 58.08% ± 1.58% and that of HL-1 cells in the CQ/PLGA group was 21.52% ± 1.46% (vs. the ISO group, ***p < 0.001). The apoptosis rate of HL-1 cells in the US + CQ/PLGA group was 10.90% ± 0.68% (vs. control group, **p < 0.01, vs. other groups, ^##^ *p* < 0.01; Figures 8A,B). Therefore, compared with CQ, CQ/PLGA had better biocompatibility and could improve the effect of CQ. When the sound pressure intensity was 100 mvpp and the action time was 30 s, the cavitation effect can occur in CQ/PLGA. The increased permeability of HL-1 cells further enhanced the inhibition of ferroptosis in HL-1 cells. Therefore, US combined with CQ/PLGA could effectively inhibit ferroptosis in HL-1 cells.

## Discussion

Ferroptosis is a recently discovered form of cell death caused by peroxidation induced by the accumulation of intracellular lipid reactive oxygen species in an iron-dependent manner (Wu et al., 2021). Erastin could mediate ferroptosis through a variety of molecules including the cystine-glutamate transport receptor (system XC−), the voltage-dependent anion channel, and p53 (Yang et al., 2020; Li et al., 2021; Liu et al., 2021). ISO, a synthetic catecholamine and nonselective β-adrenergic receptor agonist, was widely used in a reproducible and well-standardized preclinical model of cardiac remodeling and dysfunction mainly due to excess production of reactive oxygen species by persistent β-adrenergic stimulation (Olmo et al., 2020; Zeng et al., 2020). Previously, Liu et al. demonstrated that ferroptosis-like cell death was observed in erastin- or ISO-treated H9c2 myocytes *in vitro* and in rats with aortic banding inducing HF, characterized by reduced cell viability with increased lipid peroxidation and labile iron pool (Liu et al., 2018). Here, interestingly, similar morphological changes were observed in HL-1 cells incubated with ISO or erastin. ISO, like erastin, induces ferroptosis in HL-1 cells. At the same time, the results showed that a certain concentration of clioquinol (CQ) could inhibit ferroptosis in HL-1 cells. CQ is a ferrous chelating agent and is also known as a metal protein attenuating compound (Lv et al., 2021; Xu et al., 2021). After entering the cells, CQ binds to Fe^2+^, which blocks the occurrence of Fenton reaction and inhibits the occurrence of ferroptosis.

Ferroptosis is a way of cardiomyocyte death, and the metal ions closely related to ferroptosis are ferrous and ferric iron (Wu et al., 2021). CQ is an antibiotic that chelates both ferrous and ferric iron and is reported to alleviate neurodegenerative diseases and cancer (Lv et al., 2021; Maheshwaran et al., 2021; Xu et al., 2021). The lipophilic nature of CQ allows for its effective use at lower concentrations (Maheshwaran et al., 2021). However, CQ has the disadvantages of low bioavailability, poor water solubility, and cytotoxicity, which limit its application (Maheshwaran et al., 2021). In recent years, with the development of nanomedicine, studies have shown that PLGA nanoparticles have the advantages of high entrapment efficiency and drug loading. At the same time, PLGA nanoparticles also have high biocompatibility and sustained-release effect (Kapoor et al., 2015; Ding and Zhu, 2018). However, PLGA nanoparticles have some disadvantages, such as strong and rigid structure, low drug loading, poor imaging, and poor ultrasonic sensitivity (Su et al., 2021).

Chen et al. developed a new type of porous lipid drug-loaded PLGA MBs, which increased the drug loading of PLGA nanoparticles, while the addition of DSPC components improved the elasticity of the shell (Chen et al., 2019). This new type of porous lipid drug-loaded PLGA MBs could oscillate under the action of low-frequency US with certain parameters, that is, the “cavitation effect,” which could increase the permeability of cell membrane and improve the efficiency of drug action (De Alwis et al., 2021; Guo et al., 2021). We chose this new type of porous lipid drug-loaded PLGA MBs as carriers to prepare lipid CQ/PLGA MBs containing CQ, which improved the shortcomings of low bioavailability, poor water solubility, and cytotoxicity of CQ (Khan et al., 2020; Lv et al., 2021).

Porous lipid CQ/PLGA MBs were prepared using a water/oil/water (W_1_/O/W_2_) double emulsification method (Chen et al., 2019). Under the action of US, porous lipid CQ/PLGA MBs produced a series of alternating changes, such as expansion, contraction, oscillation, and implosion. The increase in cell membrane permeability, that is, the “sound pore effect,” led to the better effect of CQ on HL-1 cells. Thus, we provided a new scheme to inhibit ferroptosis in cardiomyocytes and improve the cardiac function and remodeling caused by an ISO attack. However, this study has some limitations. Measuring the levels of any isoforms of arachidonate lipoxygenase (ALOX), GPX4 activity, or other antioxidants, iron content, and morphological findings would provide more relevance to ferroptosis. The clinical translation and patient compliance of the research must be investigated in the future.

## Conclusion

In summary, our experimental results showed that a certain concentration of CQ could prevent the process of ferroptosis in HL-1 cells. Lipid CQ/PLGA MBs could improve the effect of CQ. At the same time, CQ/PLGA MBs had good ultrasonic sensitivity. When the frequency was 929 kHz, the sound pressure intensity was 100 mvpp, the action time was 30 s, and the pulse cycle was 10,000. Lipid CQ/PLGA MBs could further enhance the inhibitory effect of ferroptosis on HL-1 cells.

## Data Availability

The raw data supporting the conclusions of this article will be made available by the authors, without undue reservation.
